# The Role and Mechanism of Innate Immune Regulation in Overcoming Oxaliplatin Resistance and Enhancing Anti-Tumor Efficacy in Colorectal Cancer

**DOI:** 10.3390/ph18030317

**Published:** 2025-02-24

**Authors:** Xiaoqing Wang, Meili Xi, Xing Lu, Xiangshi Tan

**Affiliations:** 1 Department of Chemistry, Fudan University, Shanghai 200433, China; 20110220119@fudan.edu.cn (X.W.); 17110220108@fudan.edu.cn (X.L.); 2 Zhongshan Hospital, Fudan University, Shanghai 200032, China; xi.meili@zs-hospital.sh.cn

**Keywords:** STING, cGAMP, immunoregulation, oxaliplatin-resistant, colorectal cancer

## Abstract

**Background/Objectives:** The reversal effect of cGAMP, as a STING pathway regulator, on oxaliplatin resistance in colorectal cancer was investigated, and its mechanism was proposed. **Methods:** The efficacy and mechanism of the cGAMP and oxaliplatin combination for oxaliplatin-resistant colorectal cancer through a nude mouse tumor model were investigated and analyzed, and a western blot analysis of tumors was applied. **Results:** The reversal effect of cGAMP on oxaliplatin resistance in colorectal cancer was investigated, and its mechanism was proposed. After OXA treatment, the IC50 values of HCT116 and HCT116/L cells were 9.04 μmol/L and 47.04 μmol/L, respectively. In nude mouse tumor models, the combination of cGAMP and oxaliplatin significantly reversed the resistance of oxaliplatin to primary drug-resistant HCT116/L colorectal cancer, and the tumor inhibition rate increased from 8% (oxaliplatin alone) to 60% (combination). In the HCT116 nude mouse transplanted tumor model, the combined treatment of cGAMP and oxaliplatin also showed a more significant tumor inhibition effect than oxaliplatin alone, and the tumor inhibition rate increased by 39%, indicating that cGAMP had a considerable improvement effect on oxaliplatin acquired resistance. These results fully demonstrated the synergistic effect of cGAMP and oxaliplatin. Western blot results showed that cGAMP enhanced the sensitivity of oxaliplatin-resistant tumor cells by down-regulating the expression of p-PI3K and p-AKT and up-regulating the expression of p53 protein. **Conclusions**: cGAMP, as an immunomodulator against oxaliplatin resistance, shows a potential application prospect in treating oxaliplatin-resistant colorectal cancer.

## 1. Introduction

Chemotherapy resistance is a major challenge in cancer treatment and one of the major causes of treatment failure in cancer patients. Colorectal cancer (CRC) is one of the most serious malignancies worldwide [1,2]. The majority of CRC patients are diagnosed at an advanced stage, with a 5-year survival rate of only 25–39% and an extremely high recurrence rate [3,4]. Chemotherapy is one of the main options for treating CRC. Oxaliplatin (OXA), as the third-generation platinum chemotherapy drug, is the first-line clinical treatment for CRC patients [5,6,7]. With the long-term use of OXA, CRC cells lose a key DNA damage response pathway during cancer evolution and eventually become resistant to OXA [8,9,10], which is a key factor leading to treatment failure and tumor progression [11].

The types of chemotherapy resistance are mainly divided into primary and acquired resistance [12,13]. Primary drug resistance is due to the presence of resistance genes in patients; some cancer cells are inherently insensitive to anti-tumor drugs. Acquired resistant tumor cells are initially sensitive to drugs, but after treatment, the efficacy gradually decreases, resulting in insensitivity [14]. OXA-based CRC regimens have improved survival by no more than 20% in stage III and less than 5% in patients with stage II localized cancer [15]. Patients with metastatic CRC who also respond to treatment are often hampered by the emergence of acquired drug resistance.

Drug combinations are often used to overcome resistance, and many studies have identified new drug combinations to improve treatment efficacy [16,17]. Recent research has shown that combination drug therapy can reduce chemotherapy resistance and thus improve the effectiveness of chemotherapy on CRC cells [18,19,20,21]. OXA is usually combined with 5-fluorouracil and is considered a first-line treatment for CRC [22]. It should not be ignored that the adverse reactions associated with OXA and 5-fluorouracil when used together are more common and severe than when 5-fluorouracil is used alone. Additionally, as the number of drugs used by patients increases, the ability of the drugs to suppress tumor cells decreases, and resistance may develop that makes chemotherapy ineffective. Furthermore, in CRC chemotherapy, tumor cells not only acquire resistance to specific drugs but may also develop cross-resistance to other drugs with different structures and mechanisms of action that have not been exposed [23,24]. Therefore, there is an urgent need to identify the potential mechanisms of OXA resistance and reversal or sensitization strategies.

Studies have shown that the resistance of colorectal cancer cells to apoptosis is one of the important mechanisms of their resistance to OXA [25,26]. One of the key factors is the activation of the PI3K/Akt signaling pathway, which increases OXA resistance [26], and its downstream signal molecule p53 is highly correlated with CRC resistance. It is reported that 50–70% of CRC cases have mutations or a functional loss of the p53 gene [27], and mutant p53 increases the resistance of CRC patients to chemotherapy [28,29,30]. Research on targeting the PI3K/Akt signaling pathway as a therapeutic target has been reported; for example, the PI3K/Akt signaling pathway selective inhibitor LY294002 can inhibit cell proliferation and induce cell apoptosis [31], reversing acquired chemotherapy resistance in esophageal cancer, lung cancer, and breast cancer cells [32,33]. It is worth noting that blocking AKT phosphorylation in gastric cancer cells could activate the cGAS/STING signaling pathway [34], and that tumor suppressor Tp53 is involved in the cGAS/STING cytoplasmic DNA sensing pathway, activating the innate immune response to inhibit tumor growth [35]. The cGAS-STING pathway is a promising approach to cancer immunotherapy by bridging innate immunity and adaptive immunity. STING agonists combined with OXA have been reported for colorectal cancer treatment [36,37]. It could be possible that the innate immune activator cGAMP has the potential to enhance the efficacy of OXA against OXA-resistant CRC cells, overcome OXA-induced CRC resistance, and enhance its anti-tumor efficacy. If so, is its mechanism related to the PI3K/AKT/p53 signal axis? These scientific questions are the main contents to investigate systematically in this paper.

In response to the above scientific questions, we investigate the reversal effect of cGAMP on OXA-resistant CRC cells, and we propose and elucidate its pharmacological mechanism of using a combination of cGAMP and OXA in a subcutaneous transplant tumor model of OXA-resistant mice.

## 2. Results

### 2.1. Effect of cGAMP on Primary OXA-Resistant HCT116/L Nude Mouse Tumor

**Figure 1:**
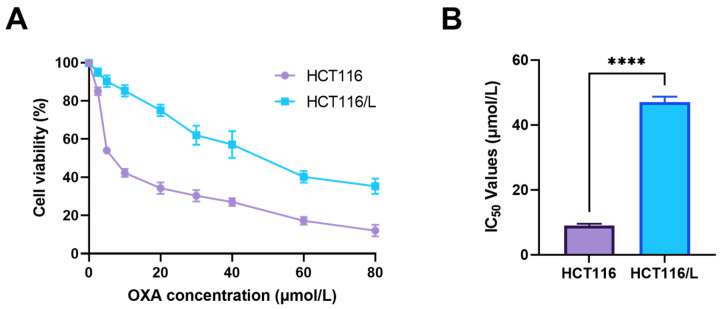
Cellular characterization of oxaliplatin-resistant HCT116/L cells: (**A**) The viability of HCT116 and HCT116/L cells treated with different concentrations of oxaliplatin for 48 h was determined through a CCK-8 assay. (**B**) The IC_50_ values of OXA in HCT116 and HCT116/L cells were measured with a CCK-8 assay. IC_50_ values in HCT116: 9.04 ± 0.86 μM; in HCT116/L: 47.04 ± 0.39 μM. RI index: 5.20. (*n* = 5; the results as shown as mean ± S.D.; ****, *p* < 0.0001).

OXA-resistant cells HCT116/L were cultured and assayed as described below, according to the literature method [38]. HCT116 cells were exposed to a culture medium with a final concentration of 2 μg/mL OXA until resistance was developed. Then, the cell proliferation activity and IC_50_ were detected under different OXA concentrations, and the degree of drug resistance of HCT116/L was calculated. As shown in Figure 1A, OXA treatment was dose-dependent on the activity of HCT116 and HCT116/L cells. When HCT116/L cells were exposed to the same concentration of OXA, their cell viability was higher than that of HCT116 cells. In addition, the cell viability of HCT116/L cells and HCT116 cells was measured after OXA treatment for 4 days (Supplementary Figure S1). Compared with the untreated OXA group, HCT116 cell viability decreased to 44% after 4 days of OXA exposure. However, HCT116/L cells showed no significant difference, indicating that HCT116/L cells could continue to grow after being released from OXA maintenance culture, and the drug resistance characteristics were stable. Based on the data on the drug concentration and inhibition rate, a nonlinear regression model was used to calculate the IC_50_ value. Figure 1B shows that, after OXA treatment for 48 h, the IC_50_ value of HCT116 was 9.04 μmol/L, and the IC_50_ value of HCT116/L is 47.04 μmol/L. The resistance index (RI) = Drug-resistant cell IC_50_/parent cell IC_50_. The RI index of HCT116/L was 5.20. The results confirmed that HCT116/L was moderately resistant to OXA, indicating that we successfully established OXA-resistant HCT116/L cells.

**Figure 2:**
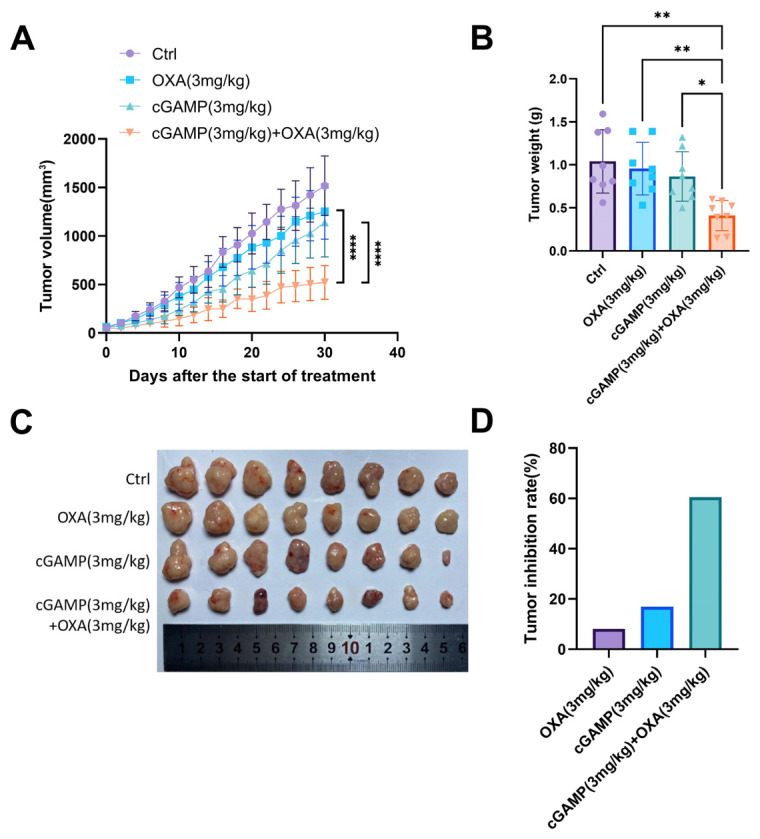
Anti-tumor effect in the HCT116/L colorectal cancer model treated with different formulations in vivo. (**A**–**D**) The tumor growth curve, average tumor weight, tumor picture, and tumor inhibition rate of mice after multiple treatments (*n* = 8; results are shown as mean ± S.D.; *, *p* < 0.05; **, *p* < 0.01, ****, *p* < 0.0001).

To systematically investigate the role of cGAMP against OXA resistance, we first established a primary OXA-resistant HCT116/L subcutaneous graft tumor model. Nude mice were randomly divided into 4 groups: a control group, a cGAMP (3 mg/kg) group, OXA (3 mg/kg) group, and a cGAMP (3 mg/kg) + OXA (3 mg/kg) group. Treatment was initiated after the tumor volume reached 50 mm^3^. The drug was administered intraperitoneally once every 2 days, and changes in tumor volume were monitored during treatment. After 30 days of administration, the nude mice were euthanized, the tumors were collected, and the tumor tissues were analyzed. As shown in Figure 2A, after 30 days of administration, the combined administration of cGAMP and OXA had the best effect on tumor inhibition compared with the control group. There were significant differences between the cGAMP and OXA in both monotherapy and the combination treatment group. The combined treatment group had the smallest tumor weight and was significantly different from the two monotherapy groups, while there was no significant difference between the two monotherapy groups and the control group (Figure 2B,C). The statistical tumor suppression rate of the drug administration group (Figure 2D) showed that the tumor suppression rate of the OXA monotherapy group was 8%, that of the cGAMP monotherapy group was 16%, and that of the combined treatment group was 60%. These results indicated that, in OXA-resistant HCT116/L tumor-bearing nude mouse, cGAMP combined OXA showed a powerful synergistic effect, significantly enhancing the anti-tumor effect and reversing OXA resistance to CRC.

In addition, we found that the combination of cGAMP and OXA still had a significant therapeutic effect and synergistic effect at a very low cGAMP dose (1 mg/kg), although cGAMP (1 mg/kg) itself could not show an obvious antitumor effect. As shown in Supplementary Figure S2, the tumor inhibition rate of OXA alone was 12%, and the combination (cGAMP 1 mg/kg, OXA 3 mg/kg) was 55%. Tumor inhibition rates of 1, 3, and 9 mg/kg cGAMP were 3%, 16%, and 34%, respectively, indicating a significant dose-response relationship. The results also fully demonstrated the synergistic effect of cGAMP and OXA (1 + 1 > 2).

### 2.2. Effect of cGAMP on Acquired OXA-Resistant HCT116 Tumor of Nude Mice

To investigate the effect of cGAMP combined with OXA on overcoming OXA-acquired drug resistance, we established a nude mouse model of HCT116 subcutaneous tumor transplantation. We assigned the nude mice randomly to four groups: the model control, cGAMP (5 mg/kg), OXA (3 mg/kg), and cGAMP (5 mg/kg) + OXA (3 mg/kg). Treatment was initiated after the tumor volume reached 50 mm^3^. We administered the drug into the abdominal cavity every two days, and we monitored tumor volume changes during treatment. After 30 days of administration, the nude mice were euthanized, blood and tumors were collected, and tumor tissue was weighed and photographed.

**Figure 3:**
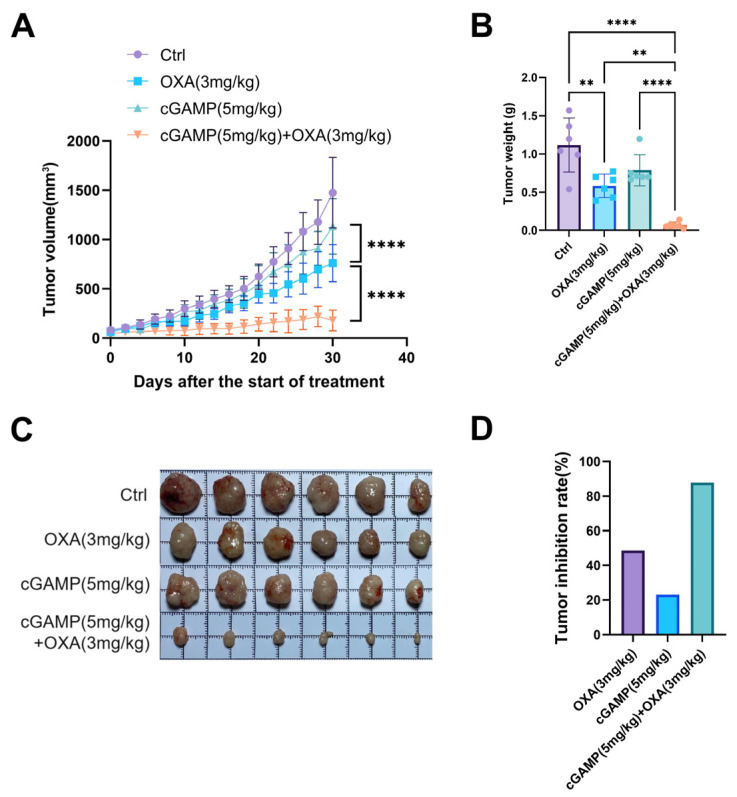
Anti-tumor effect in the HCT116 colorectal cancer model treated with different formulations in vivo. (**A**–**D**) The tumor growth curve, average tumor weight, tumor picture, and tumor inhibition rate of mice after multiple treatments (*n* = 6; results as shown as mean ± S.D.; **, *p* < 0.01; ****, *p* < 0.0001).

As shown in Figure 3A, at 30 days of administration, the combined cGAMP and OXA group had the smallest mean tumor volume compared to the model control group, followed by the OXA group. There was a significant difference in tumor volume between the cGAMP group and the OXA group. There was a significant difference in tumor volume between the combined treatment group and the OXA administration group. The tumors were removed, weighed, and photographed for observation. The results of each treatment group were consistent with the tumor volume results, and there were significant differences between the combined treatment group and the cGAMP alone group or the OXA alone group (Figure 3B,C). The statistical tumor suppression rate of the drug administration group (Figure 3D) showed that the tumor suppression rate of the OXA monotherapy group was 48%, that of the cGAMP monotherapy group was 23%, and that of the combined treatment group was 87%. These results indicate that cGAMP combined with OXA can significantly enhance the anti-tumor effect in an HCT116 tumor-bearing nude mouse model compared with monotherapy, indicating that the combination of cGAMP and OXA also has an obvious synergistic anti-tumor effect on the OXA-resistant HCT116 tumor model in nude mice.

**Figure 4:**
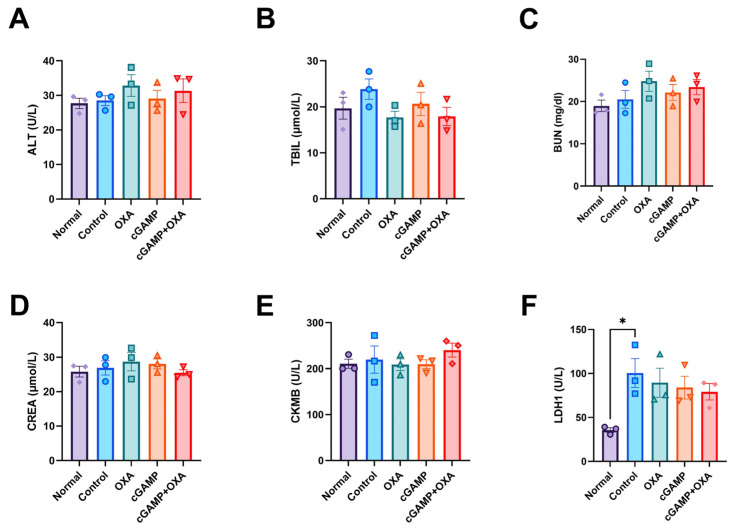
Serum biochemical test of mice. Hematological analyses of ALT (**A**), TBIL (**B**), BUN (**C**), CREA (**D**), CKMB (**E**), and LDH1 (**F**) were evaluated in each HCT116 xenografted colon cancer model mouse group. (*n* = 3; the results are shown as mean ± SEM; *, *p* < 0.05). ALT, alanine transaminase; TBIL, total bilirubin; BUN, blood urea nitrogen; CREA, creatinine; CKMB, creatine kinase isoenzyme MB; LDH1, lactate dehydrogenase 1.

Furthermore, we conducted a preliminary safety test of the combined administration of cGAMP and OXA, and we centrifuged the anticoagulant blood of 4 groups of nude mice with HCT116 subcutaneously transplanted tumors, respectively, and collected supernatant plasma. Biochemical methods were used to analyze the effects of the cGAMP and OXA combined therapy on liver function indexes (ALT and TBIL), kidney function indexes (BUN and CREA), and cardiac function indexes (CKMB and LDH1) (Figure 4). The results showed that these indicators were within the normal range, and there was no significant difference compared with the control group, indicating that cGAMP and OXA combined therapy did not have adverse effects on liver, kidney, or heart function.

### 2.3. The Pharmacological Mechanism of cGAMP-Reversing OXA Resistance in Colorectal Cancer

The phosphatidylinositol 3-kinase/protein kinase B (PI3K/AKT) pathway has been investigated extensively to determine its role in drug resistance and driving malignancy processes in solid cancer patients [39,40,41,42,43]. EVA1A, a tumor suppressor, has been reported to reverse lenvatinib resistance in liver cancer by modulating the PI3K/AKT/p53 signaling axis [44]. cGAMP inhibits the metastasis of triple-negative breast cancer by inhibiting the PI3K/AKT signaling pathway [45]. In addition, AKT promotes the phosphorylation of MDM2, which leads to p53 degradation, the regulation of the cell cycle, or the inhibition of tumor cell apoptosis [46,47]. Downregulated expression of p53 often leads to the resistance of tumor cells to platinum drugs [48,49]. However, in the case of OXA resistance, whether cGAMP activates p53 remains unknown. Given the key roles that the PI3K/AKT/p53 signaling axis and cGAMP may play in tumor therapy, we conclude that the PI3K/AKT/p53 signaling axis and cGAMP may perform important functions in reversing OXA resistance to CRC.

**Figure 5:**
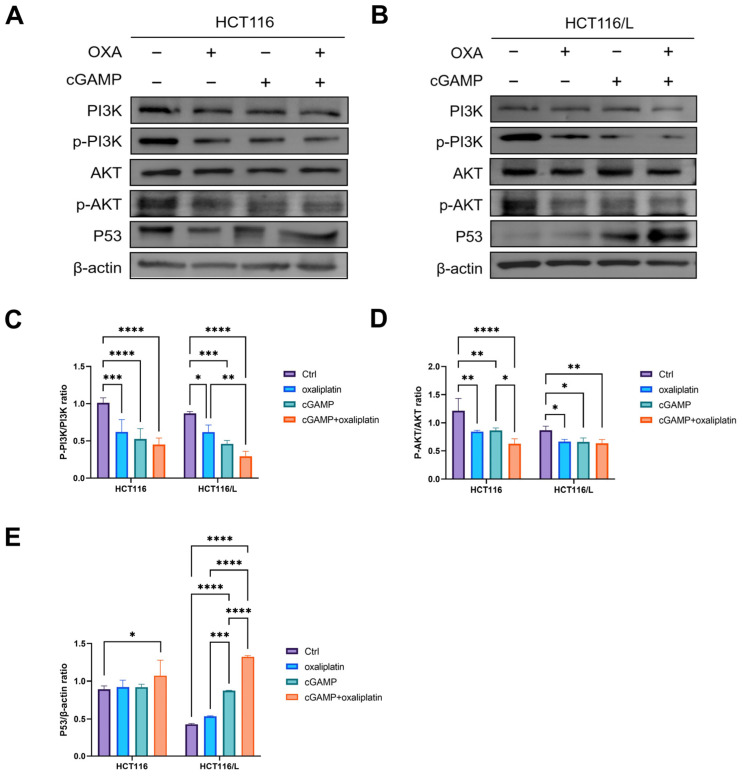
Western blot revealed the PI3K/AKT pathway and P53 protein expression level in each xenografted colon cancer model mouse group. HCT116/L: control group, cGAMP (3 mg/kg) group, OXA (3 mg/kg) group, cGAMP (3 mg/kg) + OXA (3 mg/kg) group. HCT116: control group, cGAMP (3 mg/kg) group, OXA (3 mg/kg) group, cGAMP (3 mg/kg) + OXA (5 mg/kg) group. (**A**,**B**) Quantification of PI3K, P-PI3K, AKT, P-AKT, and P53 protein expression levels in HCT116 and HCT116/L model mice. (**C**–**E**) Quantitative analysis of protein expression in each group. (*, *p* < 0.05; **, *p* < 0.01; ***, *p* < 0.001; ****, *p* < 0.0001).

To verify this inference, we performed western blot tests on the nude mouse tumor tissues studied above (Figure 5A,B). In the HCT116 nude mouse tumor model, compared with the model control group, the phosphorylation levels of PI3K and AKT were significantly down-regulated in the cGAMP group alone, while the changes in p53 were not significant. However, when cGAMP and OXA were combined, the phosphorylation levels of PI3K and AKT were significantly down-regulated, and the expression level of p53 was significantly up-regulated (Figure 5C–E). This indicated that, in the acquired drug-resistant HCT116 nude mouse tumor model, p53 activity was not effectively activated during cGAMP monotherapy, and its ability to reverse drug resistance was limited, which was consistent with the results of tumor suppression in vivo (Figure 3D). However, the combination of cGAMP and OXA can jointly inhibit the PI3K/AKT signaling pathway, play a synergistic role in increasing p53 expression, significantly increase the sensitivity of OXA to tumor cells, and effectively reverse OXA drug resistance.

In the primary HCT116/L nude mouse tumor, compared with the control group, the phosphorylation levels of PI3K and AKT in the cGAMP administration group were significantly down-regulated (47%, 24%), and the expression level of p53 was significantly up-regulated by a factor of 1. Surprisingly, compared with the control group, when cGAMP and OXA were combined, the phosphorylation levels of PI3K and AKT were significantly down-regulated (66%, 27%), and the expression level of p53 was significantly up-regulated by a factor of 2. This indicates that, in the primary OXA-resistant HCT116/L tumor model, cGAMP can significantly and effectively inhibit the PI3K/AKT signaling pathway, thereby increasing p53 expression, and the effect is more obvious when combined with OXA (Figure 5C–E), which is consistent with the above anti-tumor research results.

In the HCT116 tumor model, compared with the control group, the phosphorylation levels of PI3K and AKT were decreased in the OXA administration group, but the expression levels of p53 were not significantly changed. This result is consistent in the HCT116/L model (Figure 5C–E). This indicates that, although OXA can inhibit the PI3K/AKT signaling pathway to a certain extent, the expression of p53 is inhibited. On the one hand, this result indicates that the PI3K/AKT/p53 signaling axis does affect tumor OXA resistance, which is consistent with the literature [43,44]. On the other hand, it confirmed our inference that p53 protein expression is inhibited in the case of drug resistance. When administered in combination with OXA, cGAMP activates p53 or reduces mutant p53 through autophagy, thereby promoting tumor cell apoptosis and inhibiting CRC tumor growth and OXA resistance. However, the ability of cGAMP to activate p53 was different in the primary and acquired OXA-resistant CRC models, and it was more significantly enhanced in the primary OXA-resistant HCT116/L model when combined with OXA.

**Figure 6:**
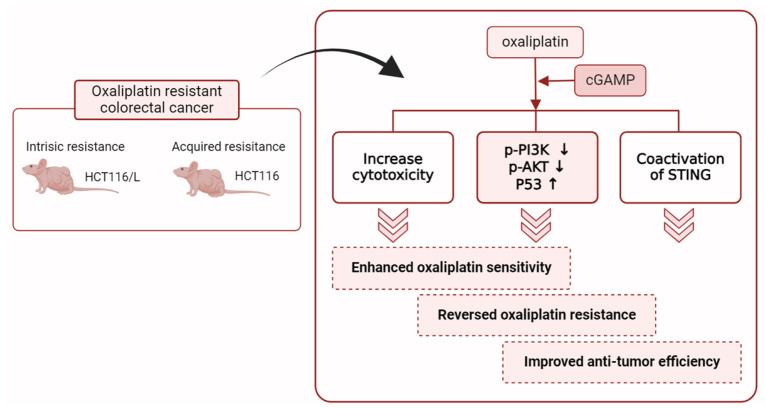
Schematic diagram shows that cGAMP and oxaliplatin synergistically enhance the treatment of colorectal cancer and reverse oxaliplatin resistance. Figure created with BioRender (https://app.biorender.com/illustrations/677e70555690f93f8eb99d72, accessed on 15 January 2025).

Based on the above findings, we proposed the mechanism by which innate immune regulation reverses OXA resistance in CRC (Figure 6). First, cGAMP induces cytotoxicity, promotes tumor cell apoptosis, and enhances OXA sensitivity. Second, cGAMP and OXA jointly inhibit PI3K and AKT phosphorylation, thereby increasing the expression level of tumor suppressor gene TP53 and reversing OXA resistance. Third, the DNA fragments caused by OXA trigger STING signaling pathway and cooperate with cGAMP to activate the immune system, produce immune factors such as IFN, and enhance anti-tumor efficiency. The study indicates that the innate immune regulatory factor cGAMP plays an important role in the anti-OXA resistance of CRC, and the specific mechanism of its reversal of OXA resistance needs to be further studied. This study provides a new idea and foundation for further research on this system.

## 3. Discussion

Oxaliplatin (OXA) is a first-line chemotherapy drug in the clinical treatment of advanced CRC and exhibits strong anti-CRC activity [50]. However, almost all patients develop resistance after long-term treatment with OXA, which limits the effectiveness of treatment [51]. To overcome OXA resistance, it is usually used in combination with other chemical drugs in clinical practice, but it also increases the possibility of toxicity and multi-drug resistance. Therefore, there is an urgent need to elucidate the molecular mechanism of OXA resistance and develop reversal strategies. In this study, we demonstrated for the first time that cGAMP combined with OXA can effectively reverse OXA resistance in CRC. cGAMP itself has a certain inhibitory effect on tumors and can enhance the sensitivity of drug-resistant cells to OXA in a concentration-dependent manner. Even when a low dose of cGAMP has no obvious tumor inhibitory effect, its combination with OXA can still promote CRC sensitization and reverse OXA resistance, significantly improving the therapeutic effect of OXA. The findings of our study reveal the potential pharmacological mechanism through which cGAMP can reverse OXA resistance in colorectal cancer and increase p53 expression by inhibiting the PI3K/AKT signaling axis, thereby promoting tumor cell apoptosis and enhancing the anti-tumor immune response. This may provide a promising strategy for treating OXA-resistant CRC. Given the widespread use of platinum drugs in CRC treatment and the severity of drug resistance, the results of this study provide new ideas for studying OXA resistance and its reversal mechanism.

In recent years, a large amount of evidence has shown that cGAMP, as an agonist of interferon-stimulating gene protein STING, can activate innate immunity and release pro-inflammatory cytokines such as interferon to inhibit the proliferation and metastasis of malignant tumor cells such as colorectal cancer, breast cancer, and melanoma. In this study, the effects of cGAMP on OXA-resistant tumor cells and its mechanism were investigated. We adopted OXA primary and acquired resistant cell lines (HCT116/L and HCT116), and treated HCT116 cells and HCT116/L cells with OXA in a subcutaneous transplanted tumor mouse model to simulate the anti-tumor treatment process of acquired OXA-resistance and primary OXA-resistance. In the primary OXA-resistant HCT116/L model, the tumor inhibition effect of cGAMP and OXA was weak when treated separately, while the tumor inhibition effect of the cGAMP and OXA combined administration was significantly increased. In the absence of a single dose of cGAMP (1 mg/kg) with a significant anti-tumor effect, the combination of cGAMP and OXA still had a significant cooperative effect. This finding suggests that cGAMP increases the susceptibility of CRC to OXA and that cGAMP and OXA play a synergistic role in reversing drug resistance. This was also confirmed in the model of acquired OXA-resistant xenotransplantation.

Mechanistically, OXA inhibits the activation of the PI3K/AKT signaling axis in a variety of cancers [52], and whether this signaling is activated or suppressed is critical in determining how cells respond to drug therapy [53]. The PI3K/AKT/MDM2 signaling axis is thought to respond to growth and survival signals and shows anti-apoptotic effects in the vast majority of tumors. Studies have shown that AKT enhances the phosphorylation modification at Ser166 of MDM2 through the PI3K pathway, which can enhance the nuclear localization of MDM2 and increase the ubiquitination and degradation of p53 [54]. Several studies have reported that decreased p53 expression is associated with OXA chemotherapy resistance in CRC patients. Pothuraju et al. reported that the MUC5AC protein promotes OXA resistance by down-regulating the expression of p53 and its target gene in CRC [55]. As expected, our study found that OXA activates the PI3K/AKT signaling pathway, promoting the proteasomal degradation of p53 in both primary and acquired resistance xenografts, thereby exacerbating OXA resistance in CRC cells. cGAMP combined with OXA inhibits PI3K/AKT signaling pathway, resulting in increased p53 expression and reversing OXA resistance. Here, the role of the PI3K/AKT/p53 signaling axis in OXA resistance is consistent with its role in the development of chemical resistance, as previously reported [54,56]. Our results suggest that cGAMP combined with OXA can mediate the sensitivity of CRC cells to OXA therapy by influencing the PI3K/AKT/p53 signaling axis.

According to the above analysis, one important reason for the synergistic effect of cGAMP and OXA may be that the activation of innate immunity has a direct anti-tumor effect and enhances tumor antigen presentation in the T-assisted 1(TH1) cytokine and chemokine environment, thus promoting anti-tumor adaptive immune response. Similar studies have shown that the ability of toll-like receptor 9 (TLR9) agonists, such as CpG oligonucleotides, to increase the anti-tumor activity of cisplatin is associated with drug-induced downregulation of some DNA repair genes in tumors, thus making tumor cells more vulnerable to DNA damage [57,58]. Another important reason may be that the innate immune agonist cGAMP improves the CRC sensitivity of OXA resistance, which can overcome CRC resistance and enhance its anti-cancer efficacy.

In addition to ssDNA or dsDNA produced via tumor cells (e.g., genomic instability), chemotherapy-induced DNA damage can directly induce dendritic cell (DC) maturation and recruitment and may work synergistically with STING activation [58,59,60,61]. On the one hand, as an immunogenic cell death (ICD) inducer, OXA produces many double-stranded DNA (dsDNA) cross-links, providing enough tumor-specific antigen or tumor-associated antigen (TAA) for STING agonist-induced anti-tumor immunity, thus enabling the entire tumor to be converted into a vaccine [62], which will greatly increase the therapeutic effect. On the other hand, after STING activation, the tumor microenvironment (TME) is easily regulated and returns to the active “hot” tumor state, which is conducive to overcoming the immune escape generated via the tumor [63,64,65]. Specifically, the activation of STING in tumor cells directly induced the mode of death (apoptosis, necrosis, etc.) [66], while also increasing the sensitivity of tumor cells to cytotoxic T lymphocytes (CTLS) and eliminating inhibitory immune cells within TME [67]. In addition, cGAMP can also activate the DNA damage response in tumor cells and induce cell cycle arrest, autophagy, and apoptosis [68,69]. With TME normalized, STING agonists tended to synergistically enhance OXA’s anti-tumor effects.

Previous research on reversing OXA resistance mainly focused on small molecule inhibitors, nanocarrier sensitization, gene regulation, and combined immunotherapy. These research strategies targeted different resistance mechanisms. Small molecule targeted inhibitors can enhance the cytotoxicity of OXA and improve the treatment effect of colorectal cancer patients [70], showing great potential in reversing OXA resistance. As a representative of small molecule targeted inhibitors, PI3K/AKT/mTOR pathway inhibitors can enhance the cytotoxicity of OXA and increase the apoptosis induced via OXA [71]. However, further research is needed to optimize treatment regimens and address the development of drug resistance. Immunotherapy combined with the reversal of OXA resistance strategy provides new treatment options for colorectal cancer patients. By targeting immunosuppressive cells, modulating immune checkpoints, and enhancing the function of immune effector cells, these combined strategies can overcome drug resistance mechanisms and improve patient treatment outcomes and survival rates [12]. As a representative of immunotherapy, the STING activator cGAMP combined with OXA not only enhances cytotoxicity and promotes the immunogenic cell death of tumor cells [36] but also provides a stronger anti-tumor response through the synergistic activation of STING, which activates innate immunity. Therefore, the tumor inhibition rate of combined treatment far exceeds that of single-drug treatment. In this regard, the cGAMP combination therapy strategy is superior to the PI3K/AKT/mTOR pathway inhibitor strategy.

In this study, we found that cGAMP reverses OXA resistance in CRC by regulating the PI3K/AKT/p53 signaling axis. But this study is limited to the treatment of OXA-resistant colorectal cancer. Whether cGAMP can reverse resistance to other chemical drugs of CRC needs to be examined. In addition, the development of STING agonists has been a major focus of basic research and the pharmaceutical industry over the past decade [72]. Researchers have developed multiple STING agonists and validated them in preclinical models and clinical practice. However, the endogenous cyclic dinucleotides (CDNs) and CDN-derived STING agonists either limited biological effects or failed in clinical trials due to their metabolic instability, the permeability of cells, and rapid clearance [73,74,75]. Therefore, how to specifically activate STING in the tumor microenvironment is also a key issue that needs further consideration.

## 4. Materials and Methods

### 4.1. Materials

Oxaliplatin was purchased from LeYan (Shanghai, China). 2′,3′-cGAMP was purchased from Hangzhou Orenstar Biomed (Hangzhou, China).

### 4.2. Cell Culture

Human colorectal cancer cell lines HCT116 and HCT116/L were purchased from MeiXuan Biotech. HCT116 was cultured in high glucose Dulbecco’s modified eagle medium (DMEM) with 10% fetal bovine serum (FBS) (Gibco, Waltham, MA, USA). Parental HCT116 cells were exposed to increasing concentrations of oxaliplatin until the cells could grow stably in an oxaliplatin environment, thereby establishing an oxaliplatin-resistant cell line, HCT116/L, which was maintained in RPMI 1640 complete medium supplemented with 2 μg/mL of oxaliplatin. The establishment of a resistance model required HCT116 cells to be exposed to oxaliplatin for 8 months and passed approximately 70 times. The proliferative activity and drug resistance index of the cells were detected after 1 month of isolation from the drug culture. These cells were incubated at 37 °C and 5% CO_2_.

### 4.3. Oxaliplatin Sensitivity Assays

HCT116 and HCT116/L cells were seeded in a 96-well plate at a density of 3.0 × 10^3^ cells/well overnight and were then exposed to various concentrations of oxaliplatin for 48 h. Cell viability was assessed with a Cell Counting Kit-8 (CCK-8, Biosharp, Hefei, China). Briefly, 10 μL of CCK-8 solution was added to each well and incubated for 1 h at 37 °C. Then, the absorbance was measured at a wavelength of 450 nm. The 50% inhibitory concentration (IC_50_) was calculated from the survival curves. Each assay was performed in triplicate.

### 4.4. Animals

This study was conducted in accordance with the Animal Experimentation Ethics Guidelines for animal experiments of The Institutional Animal Care and Use Committee at Fudan University (Shanghai, China). Male naked mice were purchased from Shanghai Slac Laboratory Animal Co. Ltd. (Shanghai, China) and housed in pathogen-free animal facilities. All experimental animals were 6–8 weeks old unless otherwise stated and housed in an environmentally controlled breeding room with a temperature of 22 ± 2 °C and a relative humidity of 70% ± 5% (Certification No. SCXK(Hu)2022–0004).

### 4.5. Xenograft Tumor Mouse Model of Colorectal Cancer

To establish an in vivo colorectal cancer mouse model, 1 × 10^7^ HCT116 or HCT116/L cells were injected subcutaneously into the right flank of male naked mice (6–8 weeks old).

### 4.6. In Vivo Anti-Tumor Effects

To assess the anti-tumor efficacy and safety, we randomly assigned mice to different groups and administered free oxaliplatin or cGAMP intraperitoneal injection every 2 days. During the treatment, tumor volume was precisely recorded using a caliper and weighing scale. The formula V = L × W × H (L = the length; W = the width; and H = the height of the tumor) was used to calculate the tumor volume. At the end of the treatment cycle, the mice were euthanized. Tumors were collected and weighed. After the tumor was treated, part of the tumor was used for western blot, and part was frozen to −80 °C.

### 4.7. Biochemical Analysis

After the experimental mice were euthanized, one of the eyeballs was rapidly removed, and blood was dripped into an anticoagulation tube. To separate the serum, the blood samples were centrifuged at 1500–2000 rpm for 15 min and stored at −20 °C until use for evaluation of biochemical markers including ALT, bilirubin T, BUN, creatinine, CKMB, and LDH1 in serum samples. All of the analyses were determined using standard routine techniques according to the instructions of the kit [76].

### 4.8. Western Blot

WB was performed as previously described [77]. Proteins in the samples were resolved via sodium dodecyl sulfate-polyacrylamide gel electrophoresis and transferred onto polyvinylidene fluoride (PVDF) membranes. The PVDF membranes were blocked with 5% bovine serum albumin (BSA) and incubated overnight at 4 °C with antibodies against PI3K (1:1000, Servicebio, Wuhan, China), P-PI3K (1:1000, Beyotime, Shanghai, China), AKT (1:1000, Servicebio, China), P-AKT (1:1000, Servicebio, China), p53 (1:1000, Beyotime, China), and ACTIN (1:3000, Servicebio, China). The membranes were washed with TBST and incubated with horseradish peroxidase-conjugated Goat anti-rabbit IgG (H&L; 1:3000, Servicebio) for 1 h. Next, the membranes were developed in a dark room containing an enhanced ECL chemiluminescence substrate. Finally, the bands were analyzed with the Image J software 1.51j8.

### 4.9. Statistical Analysis

Data were presented as the means ± standard deviations of the results obtained from our experiments. One- and two-way analyses of variance were used to make multiple comparisons. All statistical analyses were performed using the GraphPad Prism software 9.3.1. The level of statistical significance was noted as follows: *, *p* < 0.05; **, *p* < 0.01; ***, *p* < 0.001; and ****, *p* < 0.0001.

## 5. Conclusions

In this study, we present new findings that cGAMP combined with OXA can effectively increase OXA sensitivity through the PI3K/AKT/p53 signaling axis, reverse OXA resistance in CRC, and significantly enhance anti-tumor efficacy. These findings suggest that the PI3K/AKT/p53 signaling axis may mediate OXA resistance in CRC. This study provides a theoretical basis for the application of cGAMP in the treatment of OXA-resistant CRC. cGAMP has a potential clinical application in increasing OXA sensitivity, reversing OXA resistance, and improving anti-tumor efficacy for colorectal cancer.

## Data Availability

The original contributions presented in this study are included in the article/Supplementary Material. Further inquiries can be directed to the corresponding author.

## Supplementary Materials

The following supporting information can be downloaded at https://www.mdpi.com/article/10.3390/ph18030317/s1: Figure S1: The cell viability of HCT116 and HCT116/L cells was analyzed by CCK8 after treatment with or without OXA (final concentration 5 μM) for 4 days; Figure S2: The dose-response relationship of cGAMP combined with oxaliplatin in the HCT116/L animal model. Table S1: Cardiac, liver and renal function indexes of control and treatment groups in normal nude mice.

## Abbreviations

The following abbreviations are used in this manuscript:

OXA	oxaliplatin
CRC	colorectal cancer
