# Supplementary material for: The Role and Mechanism of Innate Immune Regulation in Overcoming Oxaliplatin Resistance and Enhancing Anti-Tumor Efficacy in Colorectal Cancer
Source: Pharmaceuticals (Basel). 2025 Feb 24;18(3):317. doi: 10.3390/ph18030317 (PMC11944980; doi:10.3390/ph18030317)
Supplement: Supplementary file 1 [file pharmaceuticals-18-00317-s001.zip › pharmaceuticals-3454093-supplementary.pdf]

Supplementary information

for

The Role and Mechanism of Innate Immune Regulation in Overcoming Oxaliplatin Resistance and Enhancing Anti-Tumor Efficacy in Colorectal Cancer

Xiaoqing Wang, Meili Xi, Xing Lu and Xiangshi Tan *

*Correspondence and requests for materials should be addressed to:
xstan@fudan.edu.cn

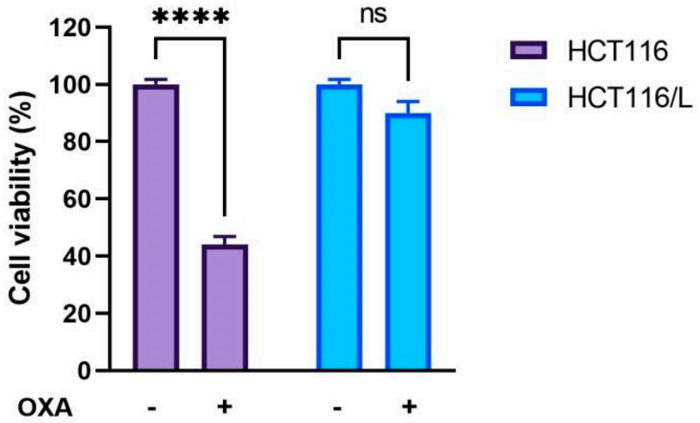

Supplementary Figure S1: The cell viability of HCT116 and HCT116/L cells was analyzed by CCK8 after treatment with or without oxa (final concentration 5 μM) for 4 days. ($n = 5$, results were shown in mean ± S.D., ****, $p < 0.0001$)

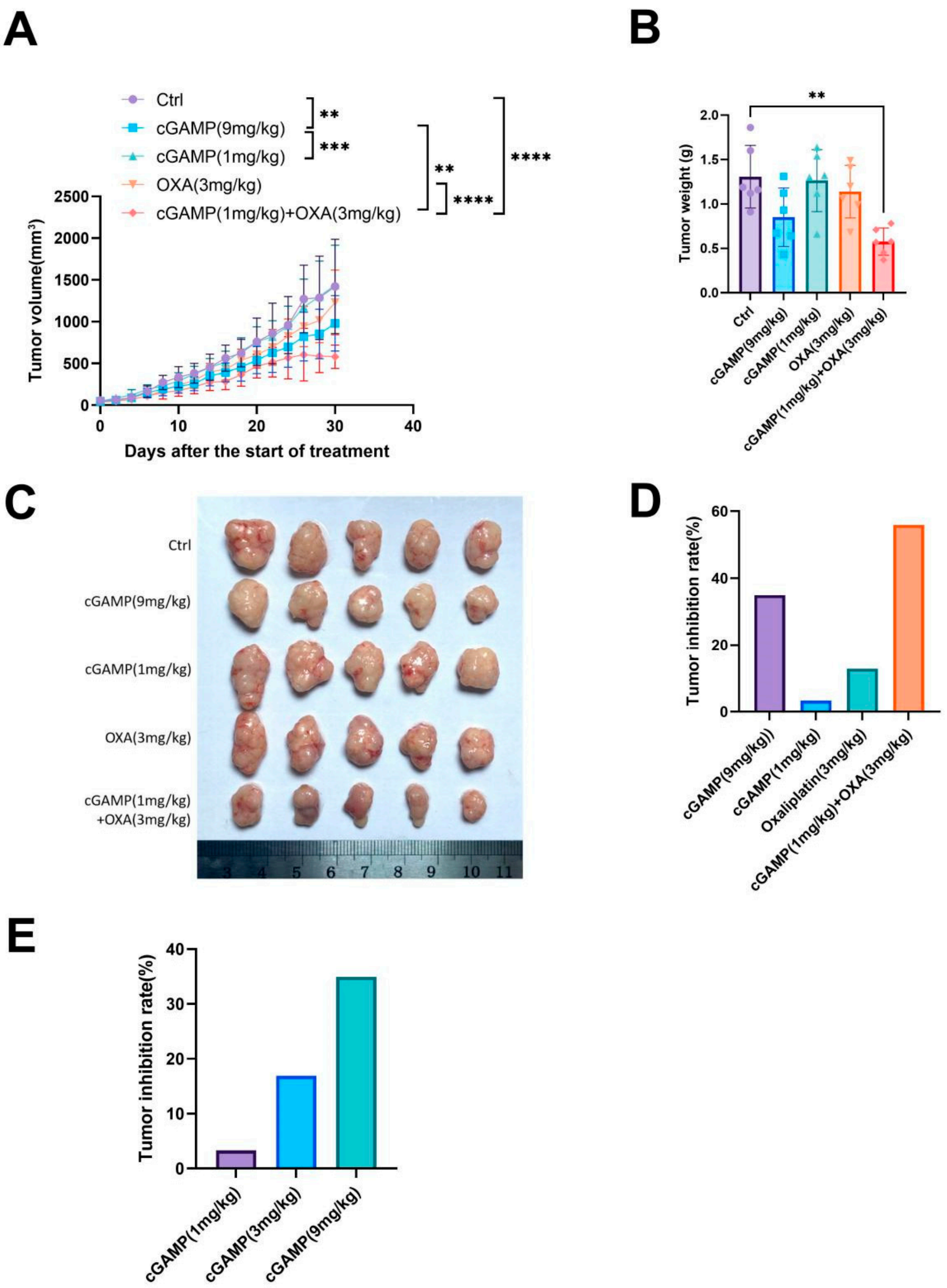

Supplementary Figure S2. The dose-response relationship of cGAMP combined with oxaliplatin in the HCT116/L animal model. A-E The tumor growth curve, average tumor weight, tumor picture, tumor inhibition rate of mice after multiple treatments (n = 5, results were shown in mean ± S.D., **, $p < 0.01$, ***, $p < 0.001$, ****, $p < 0.0001$).

Supplementary Table S1: Cardiac, liver and renal function indexes of control and treatment groups in normal nude mice.

**Groups**	**ALT (U/L)**	**TBIL (μmol/L)**	**BUN (mg/dl)**	**CREA (μmol/L)**	**CKMB (U/L)**	**LDH1 (U/L)**
Normal nude mice-Control group	25.70±2.93	20.89±4.59	18.99±2.55	25.79±3.09	210.55±29.92	35.60±4.92
Normal nude mice-Treatment group-OXA (3mg/kg) + cGAMP (3mg/kg)	25.95±5.89	19.96±5.69	23.45±4.75	25.33±2.72	219.71±27.39	28.76±8.01
